# Gas Permeation Characteristics of TiO_2_-ZrO_2_-Aromatic Organic Chelating Ligand (aOCL) Composite Membranes

**DOI:** 10.3390/membranes10120388

**Published:** 2020-12-01

**Authors:** Takashi Tachibana, Tomohisa Yoshioka, Keizo Nakagawa, Takuji Shintani, Eiji Kamio, Hideto Matsuyama

**Affiliations:** 1 Research Center for Membrane and Film Technology, Graduate School of Science, Technology, and Innovation, Kobe University, 1-1 Rokkodai, Nada, Kobe 657-8501, Japan; 187p106p@stu.kobe-u.ac.jp (T.T.); k.nakagawa@port.kobe-u.ac.jp (K.N.); shintani@port.kobe-u.ac.jp (T.S.); 2 Research Center for Membrane and Film Technology, Department of Chemical Science and Engineering, Kobe University, 1-1 Rokkodai, Nada, Kobe 657-8501, Japan; e-kamio@people.kobe-u.ac.jp (E.K.); matuyama@kobe-u.ac.jp (H.M.)

**Keywords:** TiO_2_-ZrO_2_ composite membrane, aromatic organic chelating ligand, amorphous structure, micropore, gas permeation

## Abstract

Methyl gallate (MG) and ethyl ferulate (EF) with a benzene ring were separately used as aromatic organic chelating ligands (aOCLs) to prepare two versions of TiO_2_-ZrO_2_-aOCL composite sols via hydrolysis and polycondensation reactions with titanium(IV) isopropoxide (Ti(OC_3_H_7_)_4_) and zirconium(IV) butoxide (Zr(OC_4_H_9_)_4_). Thermogravimetric and FT-IR analysis of dry gels revealed that aromatic rings were present in the residual organic matter when the gel was fired under nitrogen at 300 °C. In X-ray diffraction (XRD) measurements, the TiO_2_-ZrO_2_ composite material prepared using these two aOCLs showed an amorphous structure with no crystalline peaks for TiO_2_ and ZrO_2_. In N_2_ adsorption/desorption measurements at 77 K, the TiO_2_-ZrO_2_ samples using the aOCLs as a template appeared porous with a larger specific surface area than TiO_2_-ZrO_2_ without aOCL. TiO_2_-ZrO_2_-aOCL composite membranes were prepared by coating and firing TiO_2_-ZrO_2_-aOCL sol onto a SiO_2_ intermediate layer using an α-alumina porous tube as a substrate. Compared with the TiO_2_-ZrO_2_ membrane, the TiO_2_-ZrO_2_-aOCL membranes had higher gas permselectivity. The TiO_2_-ZrO_2_-EF membrane showed a He permeance of 2.69 × 10^−6^ mol m^−2^ s^−1^ Pa^−1^ with permeance ratios of He/N_2_ = 10.6 and He/CF_4_ = 163, while the TiO_2_-ZrO_2_-MG membrane revealed a bit less He permeance at 8.56 × 10^−7^ mol m^−2^ s^−1^ Pa^−1^ with greater permeance ratios of He/N_2_ = 61.7 and He/CF_4_ = 209 at 200 °C. A microporous TiO_2_-ZrO_2_ amorphous structure was obtained by introducing aOCL. The differences in the side chains of each aOCL could possibly account for the differences in the microporous structures of the resultant TiO_2_-ZrO_2_-aOCL membranes.

## 1. Introduction

Separation of the molecular mixtures in chemical plants is an important unit operation. This operation, however, consumes about 40% of the energy consumption of the chemical process, which makes this an important issue [1]. Technologies used in the separation of gas mixtures include distillation [2,3], adsorption [4,5], membrane separation, and so on. Among them, membrane separation has attracted attention as an energy-saving separation method because it causes no phase change during the separation procedure. A membrane-utilized separation process is expected to be applied to H_2_ recovery from petroleum refining off-gas [6], separation of normal-chain and branched hydrocarbons [7], and olefin/paraffin separation [8].

Organic polymers and inorganic materials, such as zeolite, amorphous ceramics, carbons, and metals are the materials used to produce gas separation membranes. At present, most of the separation membranes that are in practical use are organic polymer membranes because they are inexpensive and easy to manufacture [9,10]. Inorganic membranes are superior to general organic polymeric membranes in terms of thermal stability, chemical resistance, and mechanical strength, and they are expected to have utility under environments and in conditions where organic polymer membranes are of no use. [11]

Among inorganic membranes, microporous amorphous SiO_2_-based membranes [12,13,14,15] and microporous crystalline structured zeolite and metal-organic framework (MOF) membranes [16,17,18,19] have shown promise. Sol-gel-derived amorphous SiO_2_-based membranes with a pore size distribution that is well controlled show a higher level of permeance because they provide a very thin separation layer that unfortunately has a problem of low stability in the presence of water vapor. The –(Si–O–Si)_n_– polymer network that forms the pores of SiO_2_ membranes can be partially decomposed to form silanol groups under high-temperature steam conditions, and then a re-ordering of these silanol groups is followed by their polycondensation. During this process, the smaller membrane pores are densified and the larger pores are enlarged, which reduces the gas perm-selectivity performance [20]. In recent work, we focused on other ceramic materials such as TiO_2_ and ZrO_2_, which are chemically more stable than SiO_2_.

TiO_2_ and ZrO_2_ have particularly excellent chemical and physical stability, and they have been used in studies focused on nanofiltration (NF) membranes. Both TiO_2_ and ZrO_2_, however, are naturally dense materials with high crystallinity, and they can easily crystallize when calcined at high temperatures during sol-gel membrane preparation processing. The native porosity of crystalline porous materials such as zeolite and MOF increases with increasing crystallinity. Therefore, in general, their crystallinity can contribute to enhance molecular sieving performance. On the other hand, the crystalline titania and zirconia have a dense structure in which gas molecules cannot permeate, rather than a porous structure. There are ultrafiltration (UF) membranes and NF membranes made of zirconia and titania; however, these membranes are not suitable for gas separation because their porous structures are originated from the crystal grain boundaries and the size of pores are ranging from single nanometers to several tens of nanometers. Therefore, it has been difficult to form and control effective micropores for gas separation using TiO_2_ and ZrO_2_ ceramic materials, although various approaches have been attempted. Tsuru et al. succeeded in preparing a TiO_2_ NF membrane with a molecular weight cut-off (MWCO) of 500–1000 g/mol and water permeability of 0.6–1.5 × 10^−11^ m^3^ m^−2^ s^−1^ Pa^−1^ by using isopropyl alcohol (IPA) as a solvent [21]. In addition, Aust et al. have devised an amorphous structure by combining TiO_2_ and ZrO_2_, and reported that crystallization of TiO_2_-ZrO_2_ composite membranes could be suppressed even at 600 °C [22]. By using these methods, it has become possible to fabricate TiO_2_ and ZrO_2_ membranes with finely controlled microporous structures, and these have been applied to NF membranes. Anisah et al. prepared a TiO_2_-ZrO_2_ composite membrane using TiO_2_-ZrO_2_ colloidal sols for both the intermediate layer and the separation layer. The Ti/Zr ratio was changed to control the separation performance of the NF membranes. These TiO_2_-ZrO_2_ composite membranes had Ti/Zr ratios of 9/1, 7/3, 5/5 and showed water permeance ranging from 2.4 to 3.5 × 10^−3^ m^3^ m^−2^ h^−1^ bar^−1^ (LMH/bar) and MWCOs that ranged from 330 to 360 g/mol. The authors believed that the porosity of the film was increased and the number of defects was decreased with an increase in the Ti content [23].

For use as a coating material, stable and clear TiO_2_ and ZrO_2_ sols have been prepared by coordinating an organic chelating ligand (OCL) to Ti alkoxide and Zr alkoxide during hydrolysis and polycondensation reactions [24,25]. Beffer et al. prepared TiO_2_ NF and ZrO_2_ NF membranes using diethanolamine (DEA) and acetic acid as OCLs [26]. Tim Van Gestel et al. was successful in preparing a TiO_2_-ZrO_2_ composite membrane using acetylacetone (AcAc) as an OCL [27]. Sada et al. also prepared TiO_2_-ZrO_2_ composite membranes with water permeability that reached as high as 8.4–11.4 LMH/bar and an MWCO of 635–670 g by using isoeugenol (ISOH) and 2,3-dihydroxynaphthalene (DHN) as OCLs [28]. These OCLs were useful in suppressing and controlling the reactivity of metal alkoxides, which resulted in high-performance porous ceramic membranes.

A gas separation membrane prepared with TiO_2_ and ZrO_2_ materials would be considered compatible with various chemical processes. It is very difficult, however, to control pore sizes small enough for gas separations around 3 to 6 Å. In the case of silica, for example, the cristobalite crystal of silica has a dense structure consisting of a 6-membered oxygen ring with a pore diameter of 0.3 nm or less, whereas the amorphous silica structure has a denser portion, but also has a looser portion composed of 7- to 9-membered oxygen rings [29,30,31]. Those larger oxygen membered rings can give a void through which gas molecules around 0.4 nm or larger can permeate and gas selectivity is exhibited. It is also known that, even titania and zirconia, which are easily crystallized by themselves, also can have an amorphous structure by forming a TiO_2_-ZrO_2_ composite. Since it has small voids, gas molecules can permeate through it and gas selectivity can be caused [22]. However, a few studies have applied TiO_2_-ZrO_2_ composite materials to gas separation membranes. Spijksma et al. prepared a TiO_2_-ZrO_2_ composite membrane using diethanolamine (DEA) as an OCL. They observed H_2_ permeance of 3.0 × 10^−7^ mol m^−2^ s^−1^ Pa^−1^ at 200 °C with a H_2_/C_4_H_10_ permeance ratio of approximately 54 [32]. In addition, Fukumoto et al. have prepared a TiO_2_-ZrO_2_ composite membrane using isoeugenol (ISOH) as an OCL and achieved a CO_2_/N_2_ permeance ratio of approximately 46 at 35 °C [33]. A TiO_2_-ZrO_2_ composite material seems to be promising for novel microporous membranes, which can be useful for gas separation, but further studies and investigations are needed to reveal their characteristics and improve their performance.

**Figure 1:**
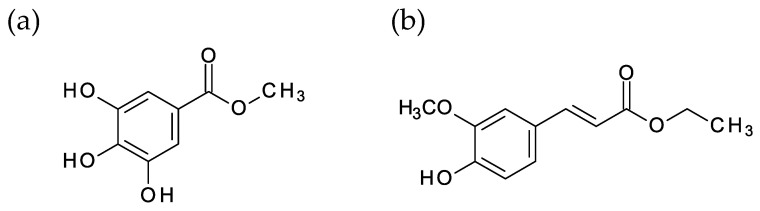
Structural chemical formulae of (**a**) methyl gallate (MG) and (**b**) ethyl ferulate (EF).

The objective of this study was to develop a TiO_2_-ZrO_2_ composite gas separation membrane using a new aromatic organic chelating ligand (aOLC) via the sol-gel method. We intended to control the pore size of the TiO_2_-ZrO_2_ composite membrane by changing the type of introduced aromatic organic chelating ligand (aOCL). From previous studies and reported papers [30,31], it was known that the organic chelating ligand with a benzene ring to form a five-membered ring with a metal element of metal alkoxide can be stably coordinated and effective for gas separation membranes. We have tried to employ various types of aOCLs with different side chain structures and coordination groups to metal element. As a result, aOCLs that had -OH or -OCH_3_ as the coordination group, and had a side chain of appropriate length whose end was neither an aldehyde group nor a carboxyl group, were suitable for preparation of gas separation membranes. We have successfully prepared microporous membranes with molecular sieving performance made of TiO_2_-ZrO_2_-aOLC composite materials. In this work, we have decided to employ such aOCLs, methyl gallate (MG) and ethyl ferulate (EF). Details of the study on inappropriate organic chelating ligands are described in the Supporting Information. Figure 1 shows the structural chemical formula of the aOCLs methyl gallate (MG) and ethyl ferulate (EF) that were used in this work.

Each of these was expected to easily form a stable five-membered ring by coordinating to Ti or Zr atoms [34]. The aromatic benzene ring structure was stable against thermal decomposition during the firing steps of the membrane preparation procedure and was expected to remain in the resultant TiO_2_-ZrO_2_ membranes as an organic residue to form an organic/inorganic nano-composite structure.

Gas separation layers were formed using TiO_2_-ZrO_2_ composite sols, which were prepared by employing MG and EF as aOCLs. We attempted to control the effective pore size for gas permeation by using the above two aOCLs with different side chains attached to the benzene ring. In this study, the difference in the structures caused by the aOCLs was discussed via X-ray diffraction (XRD) measurement and nitrogen adsorption/desorption for the TiO_2_-ZrO_2_-aOCL composite powder gel samples. The permeation selectivities of the membranes were examined via the single-gas permeation testing of several gases.

## 2. Experimental

### 2.1. Preparation of TiO_2_-ZrO_2_ Composite Sols, Gels and Powder Samples

**Figure 2:**
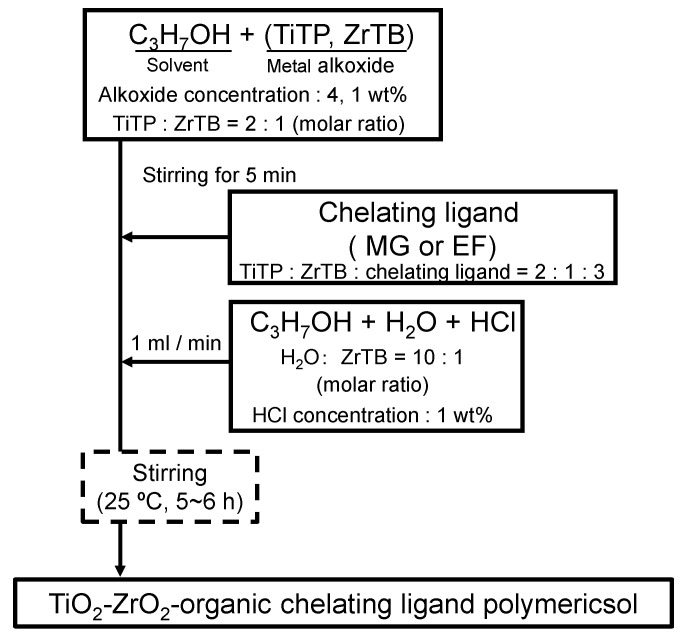
Preparation procedure for TiO_2_-ZrO_2_-aOCL sols.

TiO_2_-ZrO_2_ composite sols were prepared using Ti and Zr alkoxides. Two different types of sols can be used to prepare a sol-gel-derived TiO_2_-ZrO_2_ composite membrane [35]. One is a polymeric sol and the other is a colloidal sol. Figure 2 shows the preparation procedure for TiO_2_-ZrO_2_ composite polymeric sols. A mixture of titanium (IV) propoxide (TiTP; Aldrich, 98%, Tokyo, Japan) as a Ti alkoxide and zirconium (IV) butoxide as a Zr alkoxide (ZrTB; Aldrich, 80 wt% in 1-butanol, Tokyo, Japan) was stirred well in a solvent of 1-propanol (C_3_H_7_OH; Fujifilm Wako Pure Chemical Corporation, >99.5%, Tokyo, Japan) for 5 min. Then, an organic chelating ligand (either MG (Tokyo Chemical Industry Co., >98.0%, Tokyo, Japan) or EF (Tokyo Chemical Industry Co., >97.0%, Tokyo, Japan)) was added to the alkoxide solution. The mixture solution was additionally well stirred for 5 min again. A mixture of C_3_H_7_OH, H_2_O, and HCl as a catalyst was prepared in another pot and was added at a rate of 1 mL/min to the first mixture, followed by stirring for 5 h at room temperature under a rotation speed of 500 rpm to obtain two different TiO_2_-ZrO_2_-aOCL composite polymeric sols. The compositions of chemical reagents and solvents for preparation of the TiO_2_-ZrO_2_ composite polymeric sols are summarized in Table 1. The ratio of Ti and Zr was set at 2:1. The molar amount of aOCL was chosen to accomplish a 1:1 molar ratio of metal (Ti and Zr) to aOCL. The following chemical reactions were ideally assumed to occur during the hydrolysis of metal alkoxides and polycondensation of metal hydroxides to form the TiO_2_-ZrO_2_ composite.

$$\mathrm{Ti}{\left(\mathrm{OR}\right)}_{4}+nH_{2}O\;\to \mathrm{Ti}{\left(\mathrm{OH}\right)}_{4}+4\mathrm{ROH}\;\mathrm{Zr}{\left(\mathrm{OR}\right)}_{4}+nH_{2}O\;\to \mathrm{Zr}{\left(\mathrm{OH}\right)}_{4}+4\mathrm{ROH}\;\mathrm{Ti}{\left(\mathrm{OH}\right)}_{4}+\;\mathrm{Zr}{\left(\mathrm{OH}\right)}_{4}\to {\mathrm{TiO}}_{2}-{\mathrm{ZrO}}_{2}+4H_{2}O$$

**Figure 3:**
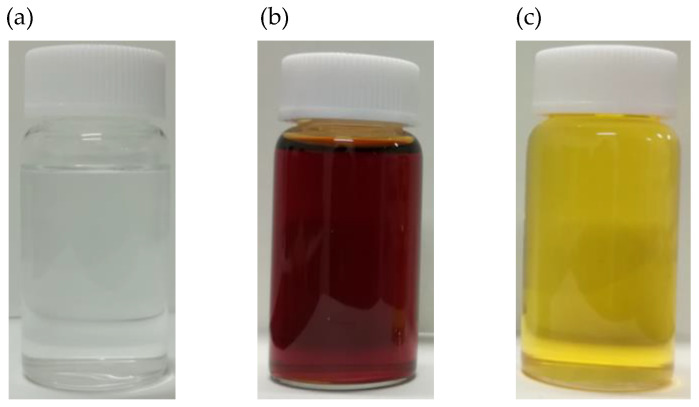
Snapshots of polymeric sols: (**a**) TiO_2_-ZrO_2_, (**b**) TiO_2_-ZrO_2_-MG, and (**c**) TiO_2_-ZrO_2_-EF.

Figure 3 features photographs of the TiO_2_-ZrO_2_ composite sols without aOCL (pure TiO_2_-ZrO_2_ sol) and with aOCL (TiO_2_-ZrO_2_-MG or TiO_2_-ZrO_2_-EF). The pure TiO_2_-ZrO_2_ composite sol was a colorless and clear solution, but when an OCL was added, the TiTP and ZrTB solutions quickly turned either red for MG or yellow for EF, but the TiO_2_-ZrO_2_-aOCL composite sols were clear. The chelating ligands were assumed to have coordinated to the Ti or Zr atoms and changed the electronic configurations of these chemicals.

In this study, gel and powder samples were prepared using the polymeric sols. These sols were dried at 40 °C for 1 day to obtain TiO_2_-ZrO_2_(-aOCL) dry gels. Then, they were fired at 500 °C under an air atmosphere to obtain TiO_2_-ZrO_2_(-aOCL) powder samples.

### 2.2. Preparation of TiO_2_-ZrO_2_ Composite Membranes

**Figure 4:**
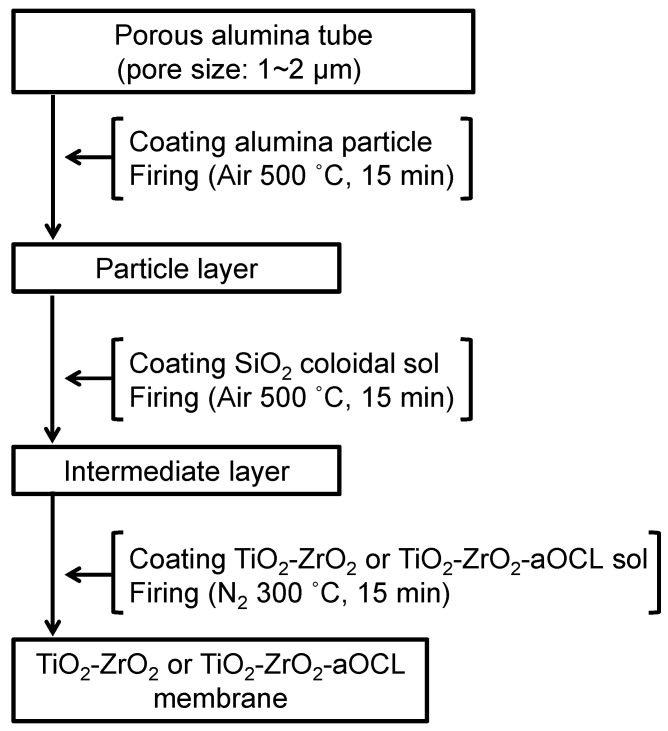
Preparation of TiO_2_-ZrO_2_(-aOCL) membranes.

A TiO_2_-ZrO_2_ composite membrane has an asymmetric multilayer structure composed of an α-alumina particle coating layer to smooth the surface of the porous α-alumina support, an intermediate layer to reduce the pore size, and an intermediate layer for separation. In this work, a SiO_2_ colloidal sol was used for the intermediate layer [36] and a coating of TiO_2_-ZrO_2_ polymeric sol formed the top separation layer. Figure 4 shows the method used to fabricate the multilayered TiO_2_-ZrO_2_ membranes. An α-Al_2_O_3_ porous tube supplied by Nikkato corp., Osaka, Japan (KU-A01; averaged pore size: 2 µm, outer diameter: 1 cm, thickness: 0.1 cm, length 5 cm), was used as the support. First, α-Al_2_O_3_ particles with mean diameters of 2 and 0.2 µm were coated onto a support at room temperature to form a particle layer. TiO_2_-ZrO_2_ polymeric sol was employed as a binder for α-Al_2_O_3_ particle coating. Then, the α-Al_2_O_3_ particle-coated support was fired at 500 °C for 15 min under air. This operation was repeated three times to obtain a smoothed α-Al_2_O_3_ support with a pore size of several hundred nanometers. Next, 0.5 wt% of SiO_2_ colloidal sol was coated onto the prepared support that was pre-heated at 180 °C to form an intermediate layer. This layer was then fired at 500 °C for 15 min under air. This operation was repeated 11 times in total to prepare an SiO_2_ intermediate layer on the α-Al_2_O_3_ support. Finally, 1 wt% of a TiO_2_-ZrO_2_ polymeric sol was coated onto the intermediate layer and pre-heated at 180 °C followed by firing at 500 °C for 15 min under air. The separation layer was produced by repeating this operation three times. In this way, a TiO_2_-ZrO_2_ composite membrane was prepared.

### 2.3. Characterization of a TiO_2_-ZrO_2_ Composite Sol and Gel Powder Samples

The particle size of the TiO_2_-ZrO_2_ polymeric sol was measured using dynamic laser scattering (DLS, ELSZ-1000, Otsuka Electronics, Osaka, Japan). The TiO_2_-ZrO_2_ polymer sol had single-nanometer-sized particles. Thermogravimetric analysis measurements were performed for the TiO_2_-ZrO_2_ gel samples. The firing temperature at which the chelating ligand would completely decompose was investigated by evaluating the behavior of the weight loss of the gel samples during the firing process by thermal gravimetric analysis (TG) (Thermo Plus EVO II, Rigaku, Tokyo, Japan). X-ray diffraction (XRD, D2 PHASER 2nd gen, Bruker, Billerica, MA, USA) profiles were measured for the TiO_2_-ZrO_2_ powder samples to detect the crystalline or amorphous structures of the powder samples. The microporous structures such as the BET-specific surface areas of the powder samples were evaluated using nitrogen adsorption/desorption isotherms at 77 K (BELSORP 28SA, Microtrac BEL, Osaka, Japan). The effects of chelating ligands on the porous structures were also discussed.

### 2.4. Characterization and Gas Permeation Measurement of TiO_2_-ZrO_2_-OCL Composite Membranes

Preparation of a crack-free intermediate layer is useful for forming a thin gas-separation layer with fewer pinholes. Therefore, characterizing, or at least checking, the pore size distribution of the intermediate layers prior to preparation of the top layer is important. Nano-perm pBETorometry [37] was used to evaluate the pore size distribution of the intermediate layer prepared in this study. An apparatus was constructed in-house to deliver a mixture of condensable vapor and non-condensable gas to a membrane. The gas permeance was reduced due to the pore plugging by the condensed vapor in the pores. Therefore, by measuring the permeance of the non-condensable gas by increasing the feeding ratio of the vapor against gas, we were able to decrease the gas permeance as a function of the relative pressure (vapor pressure divided by saturation pressure). Using the Kelvin equation, the relative pressure was easily converted to the condensation-equivalent pore diameter. The gas permeance curve can be represented by an accumulated pore size distribution mainly in the range of single nanometers. In this study, H_2_O and nitrogen were employed as the condensed vapor and non-condensable gas, respectively. An asymmetrical thin membrane structure was verified via scanning electron microscopy (FE-SEM, JSM-7500F, JEOL Ltd., Tokyo, Japan) of the membrane cross-section. The cross-section and surface of a small membrane sample piece were observed by the SEM. The sample was obtained by crushing a TiO_2_-ZrO_2_-MG membrane element whose gas permeance was actually measured by the following homemade apparatus.

**Figure 5:**
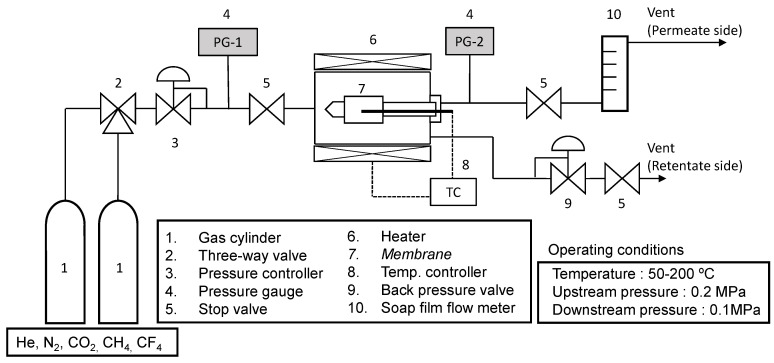
Schematic diagram of the gas permeation apparatus.

Figure 5 shows a schematic diagram of the gas permeance measurement apparatus. In order to investigate the gas permeation characteristics and pore structures of the TiO_2_-ZrO_2_-aOCL composite membranes, values for the molecular kinetic diameters and temperature dependencies of gas permeance were measured for several gas species. The upstream pressure was controlled at 0.2 MPa using a backpressure regulator, and the downstream was opened to a 0.1 MPa atmosphere. We have confirmed that the pressure resistance of the α-alumina support, the ceramic intermediate layer, and the separation layer were high enough for this measurement, and that it could withstand at least a pressure of 1 MPa in the gas phase. The gas permeation tests were performed using He, CO_2_, N_2_, CH_4_, and CF_4_, and the measurement temperature was adjusted to a range of from 50 to 200 °C. Considering the molecular sieving properties of the membranes, we selected these five types of gas molecules with different sizes, which are often used as standard for evaluating the pore structure of porous gas separation membranes. The gas permeance was measured by an automatic flow meter (SF-2U, HORIBA STEC Ltd., Kyoto, Japan) as the average value of measured five points with an error of 0.5% of read-out value after confirming that the permeation flow rate was sufficiently stable at a steady state. Therefore, these also had sufficient accuracy. In this study, only the permeation flow rate of pure gas was measured, and it depended on the set pressure and the resistance of the membrane. Therefore, the flow rate in feed side was not necessarily to be strictly controlled. Before coating the separation layer, the gas permeance of the intermediate layer was measured by using the same gas permeation apparatus. When measuring the gas flow rate of the intermediate layer, a flowmeter with a larger volume was used, and, even at a large flow rate, it was possible to measure the flow rate with a same accuracy of 0.5% of the read-out value as that of the separation layer. In order to characterize the mean effective pore size of the resultant membranes, k_0_-plot analysis was conducted for the temperature dependency of gas permeance. The details of the k_0_-plot analysis are provided in Section 3.2.3 with references.

## 3. Results and Discussion

### 3.1. Characterization of TiO_2_-ZrO_2_ Gel and Powder Samples

#### 3.1.1. Thermogravimetric Analysis

**Figure 6:**
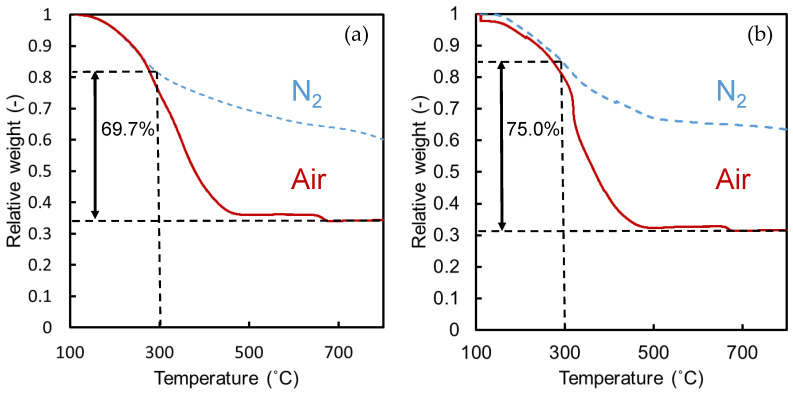
TG curves of TiO_2_-ZrO_2_-aOCL gels: (**a**) MG and (**b**) EF.

Figure 6 shows the TG curves of the TiO_2_-ZrO_2_-aOCL (MG or EF) gel samples. These curves indicate the relative weight-loss behaviors of the organic substances due to their decomposition and their removal with an elevation in temperature under air and nitrogen atmospheres. A decrease in weight occurred at around 500 °C for both gel samples during the calcination under air. The organic chelating ligands, MG and EF, were almost decomposed and removed from the remaining inorganic TiO_2_-ZrO_2_ component at temperatures below 500 °C under air.

On the other hand, the weight loss rate during elevated temperatures was slower under a non-oxidative nitrogen atmosphere than that under air. The weight residue of the gel samples under nitrogen was significantly greater even at temperatures above 700 °C, which indicated that some of the aOCL-originated organics remained in the TiO_2_-ZrO_2_ composite gels at these temperatures.

The dotted lines in Figure 6a,b indicate the ideal weight of TiO_2_-ZrO_2_ composites that was expected to remain after the complete removal of organics, as estimated from the loading amount of inorganics and organics in the starting solution. Under an oxygen-free atmosphere, the complete decomposition and removal of the organic chelating ligand would be difficult and some organics could be effectively incorporated into the TiO_2_-ZrO_2_ composite material. The residual portions of the respective organic chelating ligands introduced in the initial gels were 69.7% for MG and 75% for EF at 300 °C under nitrogen.

#### 3.1.2. IR Analysis

**Figure 7:**
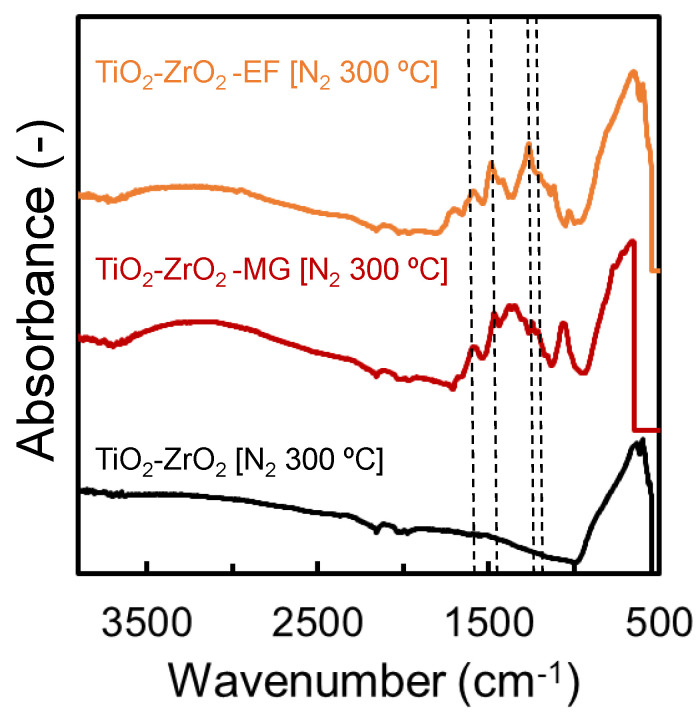
FT-IR spectrum of TiO_2_–ZrO_2_ and TiO_2_–ZrO_2_–aOCL gels calcined at 350 °C under N_2_.

Figure 7 shows the IR spectra of the TiO_2_-ZrO_2_ gel sample and the TiO_2_-ZrO_2_-organic chelating ligand (MG, EF) gel samples that were calcined at 300 °C under a nitrogen atmosphere. No peak derived from organic matter was observed in the TiO_2_-ZrO_2_ gel sample in which an organic chelating ligand was not introduced. On the other hand, in the TiO_2_-ZrO_2_-aOCL gel samples in which MG or EF was introduced, some peaks indicating the presence of a benzene ring were found at 1289, 1473, and 1579 cm^−1^. Both MG and EF have a benzene ring, as shown in Figure 1, which confirms that MG and EF were successfully introduced into the TiO_2_-ZrO_2_ material. A peak at 1267 cm^−1^ is known to indicate a coordination bond between isoeugenol (ISOH) and TiO_2_-ZrO_2_ [24]. This peak was also found in both the TiO_2_-ZrO_2_-MG and TiO_2_-ZrO_2_-EF samples. ISOH has a structure similar to that of both MG and EF, which suggests these two ligands could possibly assume a similar coordination structure, and also could likely form a coordination bond with the TiO_2_-ZrO_2_ composite.

#### 3.1.3. XRD Analysis

**Figure 8:**
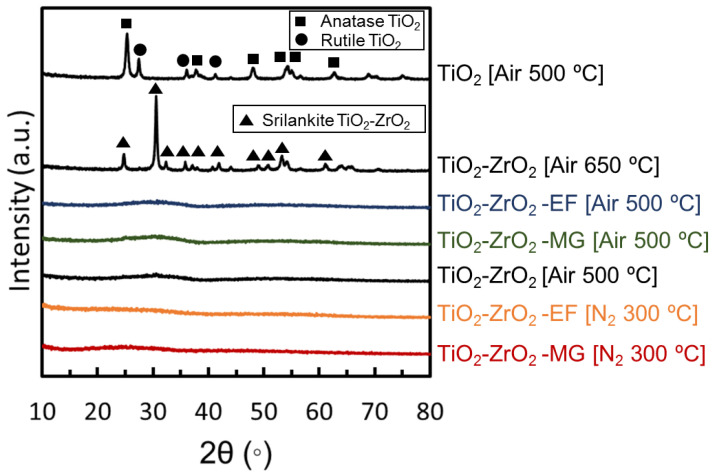
X-ray diffraction (XRD) profiles of gel powders.

Figure 8 shows the XRD profiles of TiO_2_-ZrO_2_, TiO_2_-ZrO_2_-MG, and TiO_2_-ZrO_2_-EF powder samples. Peaks of titania crystalline structures corresponding to anatase and rutile were observed for the TiO_2_ powder sample prepared at 500 °C under air as previously reported [38], while no peaks were observed for the TiO_2_-ZrO_2_ powder sample fired under similar conditions. This means that neither TiO_2_ nor ZrO_2_ crystalline structure would grow to form crystal particles sufficiently large for detection by XRD analysis. On the other hand, a powder sample calcined at 650 °C had peaks indicating the formation of a TiO_2_-ZrO_2_ composite crystalline structure of srilankite [39]. Therefore, it would be reasonable to assume that a powder composed of TiO_2_ and ZrO_2_ prepared at a temperature below the crystalizing temperature would have the amorphous structure of a TiO_2_-ZrO_2_ composite [33].

In addition, no peaks were observed for the TiO_2_-ZrO_2_-MG and TiO_2_-ZrO_2_-EF powder samples, indicating that TiO_2_ and ZrO_2_ formed a composite and had an amorphous structure that coexisted with these aOCLs. No peak was observed originating from any crystalline structure in either TiO_2_-ZrO_2_-MG or TiO_2_-ZrO_2_-EF powder samples prepared at 300 °C under a nitrogen atmosphere. As mentioned in the previous Section 3.1.1 and Section 3.1.2, under these preparation conditions, the original organics of aOCL remained in the TiO_2_-ZrO_2_ composite structures. In other words, it was possible to maintain an amorphous structure when adding an organic chelating ligand with firing at 300 °C under non-oxidation conditions, and we successfully obtained an inorganic/organic hybrid material with a TiO_2_-ZrO_2_-aOCL composite structure when using the aOCL of both MG and EF.

#### 3.1.4. Nitrogen Adsorption and Desorption

**Figure 9:**
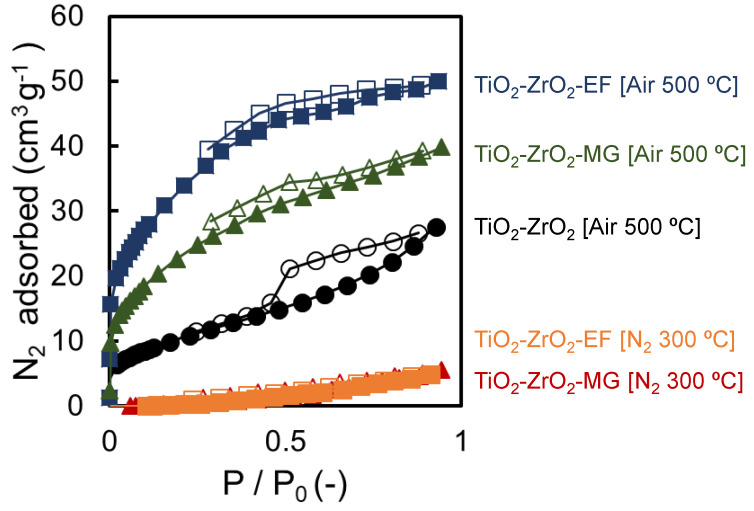
N_2_ adsorption/desorption isotherms of TiO_2_-ZrO_2_(-aOCL) powders (77 K).

Figure 9 shows the nitrogen adsorption isotherms at 77 K of TiO_2_-ZrO_2_, TiO_2_-ZrO_2_-MG, and TiO_2_-ZrO_2_-EF powder samples fired at 500 °C under air or fired at 300 °C under a N_2_ atmosphere. Nitrogen adsorption was measured at an equilibrated nitrogen relative pressure of 0.95, and the amounts along with the BET-specific surface area of each sample are summarized in Table 2. As mentioned based on the TG results shown in Figure 6, all the organic chelating ligands were decomposed and removed at 500 °C under air. The powder samples prepared with an organic chelating ligand and fired at 500 °C under air adsorbed a greater amount of nitrogen and had a larger BET-specific surface area compared with either the powder sample prepared without an aOCL or those with an aOCL fired at 300 °C under N_2_. To reiterate, as shown in Figure 6, all the organic chelating ligands were decomposed and removed at 500 °C under air. Furthermore, for the TiO_2_-ZrO_2_-MG and TiO_2_-ZrO_2_-EF powder samples fired at 500 °C, the nitrogen adsorption amount was significantly increased at low relative pressures. This suggests the presence of many micropores (pores of 2 nm or less) in those powder samples due to the formation of voids from the templating effect of the aOCLs. As a template, the coordination of an organic chelating ligand to a metal alkoxide during the sol-preparation stage could be useful for improving the micro-porosity of these types of ceramic materials [28]. We should note that the TiO_2_-ZrO_2_-EF powder sample adsorbed a larger amount of nitrogen compared with that of the TiO_2_-ZrO_2_-MG version, because EF has a slightly larger molecular size than MG, which resulted in a larger templated pore size. The adsorption/desorption isotherm of the TiO_2_-ZrO_2_ sample had a striking hysteresis, while TiO_2_-ZrO_2_-MG and TiO_2_-ZrO_2_-EF showed much smaller hysteresis loops. The mesopore ratios (2 to 50 nm) were reduced when the organic chelating ligands were introduced and more homogeneous and microporus TiO_2_-ZrO_2_ structures were formed. In the TiO_2_-ZrO_2_ sol preparation stage, the hydrolysis and condensation reaction rates were effectively suppressed by the OCLs, which formed a more homogeneous structure with fewer particle boundaries.

On the other hand, the nitrogen adsorption amount was extremely low for TiO_2_-ZrO_2_-MG and TiO_2_-ZrO_2_-EF powder samples fired at 300 °C under a nitrogen atmosphere. As shown in the TG curves and FT-IR spectrum in Figure 6 and Figure 7, respectively, the organic chelating ligand partially remained in the TiO_2_-ZrO_2_-MG and TiO_2_-ZrO_2_-EF powder samples. Molecules larger than nitrogen were expected to have very few accessible micropores due to the presence of the organic portions of aOCLs.

#### 3.1.5. SEM Observation

**Figure 10:**
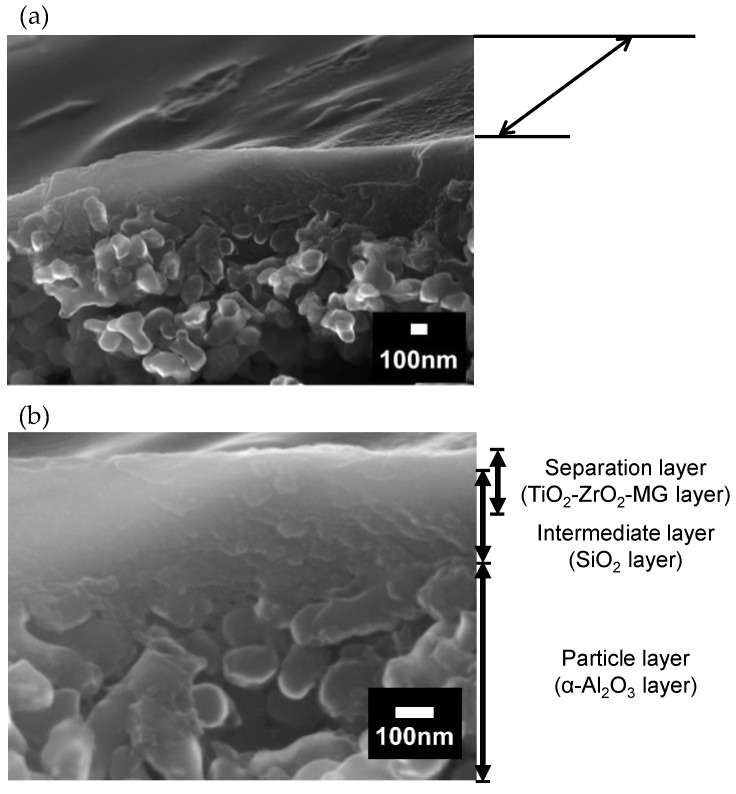
Scanning electron microscopy (SEM) images of the TiO_2_-ZrO_2_-MG membrane: (**a**) surface and (**b**) cross-section.

Figure 10 shows the SEM photographs of the surface and cross-section of the TiO_2_-ZrO_2_-MG membrane. Surface observation reveals a dense and smooth surface. The cross-sectional view shows an asymmetric multilayered membrane structure. The look of the cross-section could be divided into a particle layer fabricated by using α-alumina particles, an intermediate layer, and a very thin separation layer. The thickness of the intermediate layer was about 200 nm, which was reasonable because the intermediate layer was fabricated by coating silica colloidal particles with mean diameters of approximately 100, 50, and 30 nm. The separation layer seemed to be about 100 nm in thickness, even though the boundary between the separation layer and the intermediate layer was somewhat obscure. This might be because during the TiO_2_-ZrO_2_-aOCL polymeric sol coating procedure, some portion of the coated sol could have infiltrated the lower layer. This causes a concern that the gas permeation resistance was increased due to the formation of an unexpectedly thick separation layer, resulting in a decrease in permeance. If the separation layer can be made thinner by more strictly optimizing the pore size of the intermediate layer, it is expected to contribute to a higher level of permeability. The surface and cross-sectional views of the TiO_2_-ZrO_2_-EF membrane appear similar with no major differences.

### 3.2. Characterization of the SiO_2_ Intermediate Layer and TiO_2_-ZrO_2_ Composite Membranes

#### 3.2.1. Intermediate Layer

**Figure 11:**
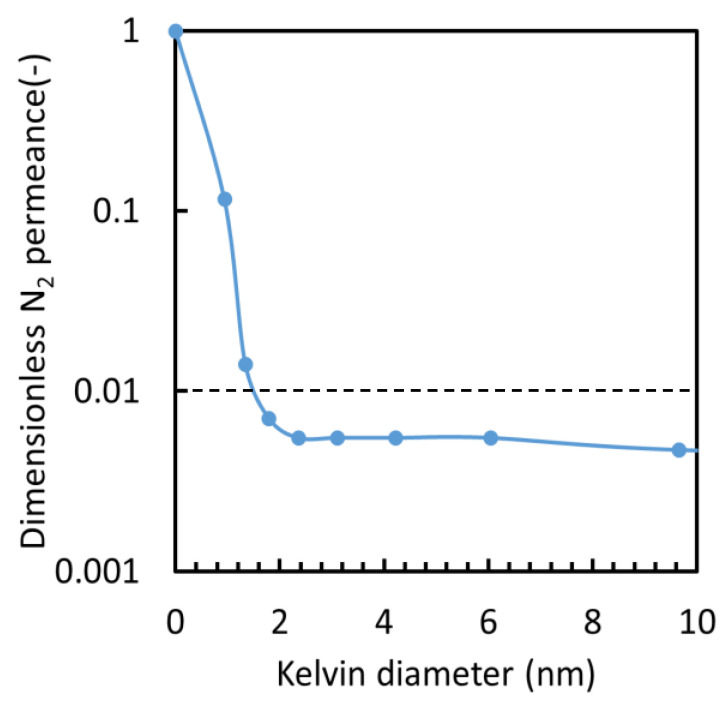
Example of the pore size distribution of the SiO_2_ intermediate layer, as measured by nanopermporometry.

Figure 11 shows a typical pore size distribution of the SiO_2_ intermediate layer prepared in this study, which was measured via nano-perm porometry. The dimensionless nitrogen permeance normalized by the nitrogen permeance at *p*/*p*_0_ = 0 was plotted against the Kelvin diameter corresponding to each relative pressure under which the non-condensable nitrogen gas permeance was measured at room temperature. The mean pore size of the distribution shown in Figure 11 was less than 1 nm, which is defined as the Kelvin diameter where the value of dimensionless gas permeance is equal to 0.5 [37]. This intermediate layer had very few pores greater than 4 nm and the leaked gas permeance ratio through such large pores was less than 0.5%, which indicates that it was free from significant pinholes. The intermediate layer shown in Figure 11 was employed for the fabrication of a TiO_2_-ZrO_2_-MG membrane. This intermediate layer could be prepared with sufficient reproducibility, and the intermediate layer membrane used for the TiO_2_-ZrO_2_-EF membrane had a pore size distribution that was similar to that shown in Figure 11, with nearly the same mean pore size and a similar dry nitrogen gas permeance, as summarized in Table 3.

#### 3.2.2. Kinetic Diameter Dependency of Gas Permeance at 200 °C

**Figure 12:**
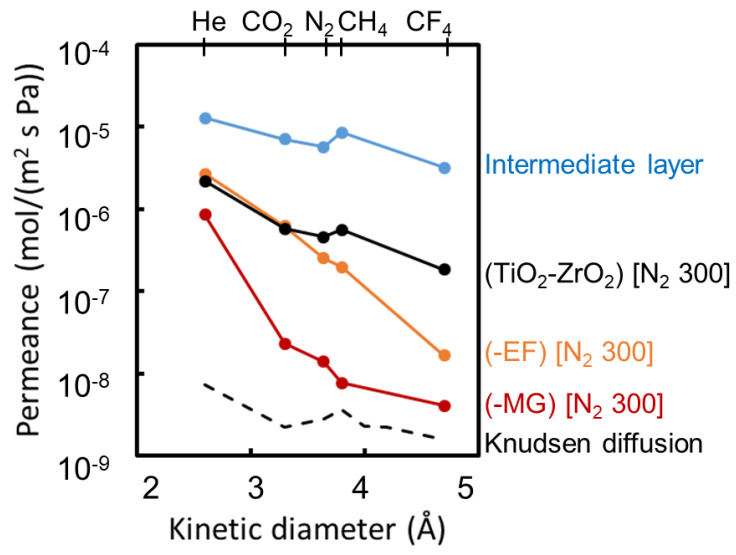
Kinetic diameter dependence of gas permeance at 200 °C.

Figure 12 shows the dependence of single-gas permeance on molecular kinetic diameter for the TiO_2_-ZrO_2_, TiO_2_-ZrO_2_-MG, and TiO_2_-ZrO_2_-EF membranes prepared by firing at 300 °C under a nitrogen atmosphere. Gas permeance data on a representative intermediate layer membrane were also plotted against the kinetic diameter for reference. For the single-gas permeation test, five gas molecules with different kinetic diameters were employed: He (0.26 nm), CO_2_ (0.33 nm), N_2_ (0.364 nm), CH_4_ (0.38 nm), and CF_4_ (0.47 nm). The broken curve in the figure indicates that Knudsen-based permeance is inversely proportional to the square root of the molecular weight. The absolute values of the Knudsen permeance are arbitrary and relative. The observed gas permeance and several gas permeance ratios for each membrane are summarized in Table 4.

The TiO_2_-ZrO_2_ composite membrane without an organic chelating ligand showed a Knudsen diffusion-like selectivity similar to that of the intermediate layer membrane. This suggests the membrane has relatively large pores because large molecules could permeate but the permeance of He was similar to that for the TiO_2_-ZrO_2_-EF membrane.

On the other hand, the TiO_2_-ZrO_2_-aOCL membranes fired at 300 °C under a nitrogen atmosphere showed much higher gas molecular permselective performances of He/N_2_ = 10.6 and He/CF_4_ = 163 for the EF version and He/N_2_ = 61.7 and He/CF_4_ = 209 for the MG version. The thermogravimetric curves in Figure 6 and the IR spectrum in Figure 7 suggested that the improved molecular sieving was due to the sources of residual organic matter. In the aOCL-free TiO_2_-ZrO_2_ composite membrane, gas molecules dominantly permeated the relatively larger pores, which could be attributed to the grain boundary pores, as predicted by the nitrogen adsorption/desorption analysis in Figure 9. For TiO_2_-ZrO_2_-aOCL membranes, molecular sieving was improved due to the formation of a more homogeneous TiO_2_-ZrO_2_ structure with fewer grain boundary pores. Even if a small amount of larger pores remained, they could have been filled and plugged by the residual organic matter. As shown by the results of the nitrogen adsorption/desorption isotherms for the TiO_2_-ZrO_2_-aOCL samples in Figure 9, the porosity detected by nitrogen seemed to be very low, which is in good agreement with the low level of nitrogen permeance. On the other hand, the amorphous TiO_2_-ZrO_2_ structure prepared with aOCL could be effective for the permeation of He, which has a molecular size that is smaller than N_2_. This improved microporosity of the TiO_2_-ZrO_2_ framework by introducing aOCL would be expected to contribute to maintaining a high level of He permeance. The gas permeance ratios for each membrane are summarized in Table 4. The difference between gas permeation characteristics between TiO_2_-ZrO_2_-MG and TiO_2_-ZrO_2_-EF membranes are discussed later in Section 3.2.3 from the view point of their pore size distribution and affinity with CO_2_ or CF_4_.

**Figure 13:**
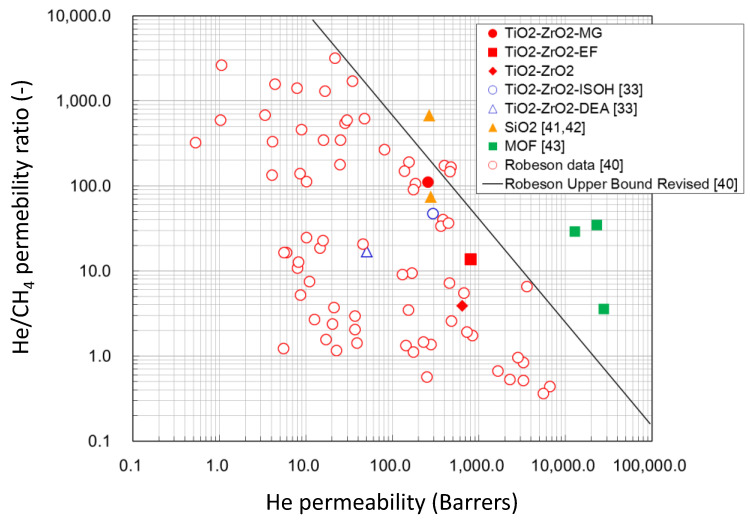
Comparison of membrane performance for He/CH_4_ in the Robeson plot.

When the thickness of the separation layer was assumed to be 100 nm at the thinnest from the SEM photograph, the helium permeability and He/CH_4_ permeability ratio at 200 °C were compared with other types of membranes in the Robeson plot [40] as shown in Figure 13. Both the TiO_2_-ZrO_2_-MG and the TiO_2_-ZrO_2_-EF membrane performances are lower than those of the silica-based membranes [41,42] and MOF membranes [43]. However, when compared with the previously reported TiO_2_-ZrO_2_-ISOH and TiO_2_-ZrO_2_-DEA membranes [33] and Robeson data for conventional organic polymer-based membranes, the performances of TiO_2_-ZrO_2_-aOCL membranes in this work were close to their upper boundary.

#### 3.2.3. Temperature Dependence of Gas Permeance

The gas translational (GT) model is based on the thermal diffusive movement of gas molecules and on the apparent activation energy, and it describes the gas permeation characteristics through the micropores of ceramic membranes. According to the GT model, permeance of the *i*-gas molecule, *P_i_*, is given by Equation (1) [36,44], where *k*_0,*i*_ is a constant that depends on the membrane structure (and also on the permeating molecular size for a modified GT model [45]), *M_i_* is the molecular weight, *R* is the gas constant, *T* is the absolute temperature, and *E*_P,*i*_ is the apparent activation energy composed of actual activation energy and adsorption energy. In the model, both the diffusivity and adsorbability of the gas molecule is taken into account. We have not calculated the diffusion and adsorption coefficient separately because the contribution of adsorption is not so remarkable and it was difficult to estimate accurate adsorption coefficient. Since the effect of diffusivity depending on molecular size, molecular weight and micropore size was more significant on gas permeance, we have analyzed obtained gas permeance based on the GT model.

(1) $$P_{i}=\frac{k_{0,i}}{\sqrt{M_{i}RT}}\exp\left(-\frac{E_{P,i}}{RT}\right)$$

**Figure 14:**
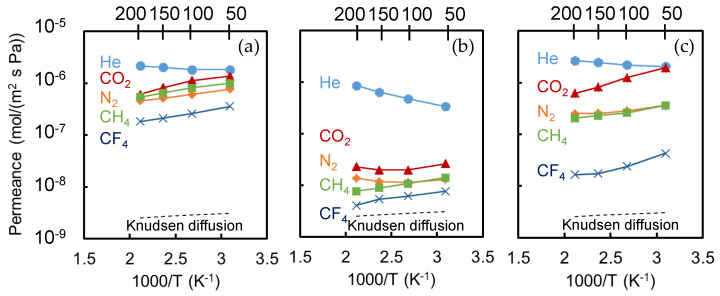
Temperature dependencies of gas permeance for three different membranes: (**a**) TiO_2_-ZrO_2_; (**b**) TiO_2_-ZrO_2_-MG; and, (**c**) TiO_2_-ZrO_2_-EF.

Significant information concerning gas permeation characteristics and membrane pore size can also be obtained by adjusting the *k*_0,*i*_ and *E*_P,*i*_ to fit Equation (1) and applying it to the temperature dependence data of gas permeance. Figure 14 shows the dependence that temperature exerts on the gas permeance of TiO_2_-ZrO_2_, TiO_2_-ZrO_2_-MG, and TiO_2_-ZrO_2_-EF membranes at temperatures ranging from 50 to 200 °C. The broken curve in the figure indicates that the Knudsen-based permeance is inversely proportional to the square root of the absolute temperature. The absolute values of the Knudsen permeance are arbitrary. The values for the resultant apparent activation energy, *E*_P,*i*_, of each gas on these membranes are summarized in Table 5.

With respect to gas permeation through the OCL-free TiO_2_-ZrO_2_ membrane, the temperature dependence of most gas molecules, except for that of He, showed a slope that was similar to that of the Knudsen permeance. In addition to the dependence of permeance shown in Figure 12 for the molecular species (*M_i_*), the gas permeation characteristics indicated that this membrane showed only Knudsen permeation and separation. An activated diffusion-like permeation for He was suggested, however, by the slight increase in permeance with an elevation in temperature. This means that even the OCL-free TiO_2_-ZrO_2_ membrane could have some small pores effective only for the permeation of He possibly due to its amorphous TiO_2_-ZrO_2_ composite structure.

The permeance of He through the TiO_2_-ZrO_2_-MG membrane showed a tendency toward activated permeation, which indicated that He dominantly permeated the small voids of the TiO_2_-ZrO_2_-MG matrix via an activated diffusion mechanism. CO_2_ and N_2_ permeance was improved with increases in the temperature above 100 °C, and also were increased with a decrease in the temperature that approached 50 °C. For the permeation of these gas molecules through the TiO_2_-ZrO_2_-MG membrane, both the activated diffusion in small pores and the Knudsen or surface diffusion in pores that were a bit larger might have competitively occurred. At high temperatures, the former mechanism would be dominant; at lower temperatures, the latter mechanism would effectively contribute to the overall permeance. The CH_4_ and CF_4_ permeation mechanisms were similar to Knudsen’s. Therefore, CH_4_ and CF_4_ mainly permeated a small number of larger pores in the membrane.

For the case of the TiO_2_-ZrO_2_-EF membrane, He exhibited activated diffusion, while N_2_ and CH_4_ permeance showed the typical temperature dependency of Knudsen diffusion. On the other hand, the permeance of CO_2_ and CF_4_ apparently increased with a decrease in the temperature, and the slopes were greater than that of Knudsen permeation. This means that CO_2_ and CF_4_ had an attractive interaction with the membrane and were able to permeate through the pores on the membrane based on the so-called surface diffusion mechanism [36].

**Figure 15:**
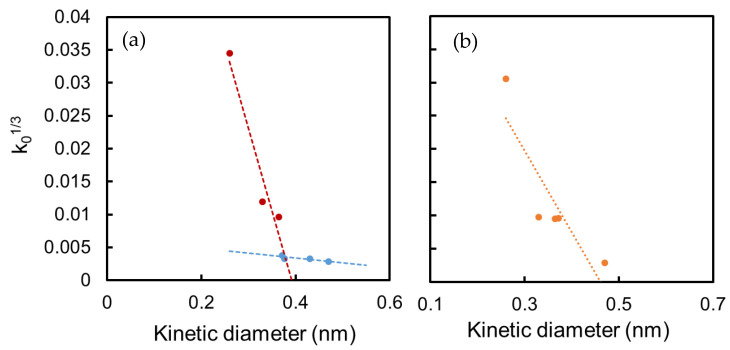
*k*_0_^1/3^ plot of (**a**) TiO_2_-ZrO_2_-MG and (**b**) TiO_2_-ZrO_2_-EF membranes.

In order to elucidate the difference in microporous structures between the -MG and -EF membranes, we conducted *k*_0_-plot analysis [45] to evaluate the micropore sizes of those membranes. In Figure 15, the values for *k*_0,*i*_^1/3^ were plotted against the kinetic diameter of a gas molecule, *i*, for TiO_2_-ZrO_2_-MG and TiO_2_-ZrO_2_-EF membranes. According to the modified GT model, in which the effective diffusion distance depends on the permeating molecular size, *d_i_*, *k*_0,*i*_ is expressed as a function of *d_i_* by Equation (2), where *L* is the thickness of the separation layer, *τ* is the tortuosity, *ε* is the porosity, and *d*_P_ refers to pore size [46].

(2) $$k_{0,i}=\frac{\varepsilon }{3\tau L}\frac{{\left(d_{P}-d_{i}\right)}^{3}}{d_{P}^{2}}\sqrt{\frac{8}{\pi }}=a{\left(d_{P}-d_{i}\right)}^{3}$$

Therefore, *k*_0_,*_i_*^1/3^ is proportional to *d_i_*, and the mean pore diameter, *d*_P_, can be given as the intercept of the *k*_0_-plot line with the *d_i_*-axis. The values for *k*_0,*i*_, obtained by fitting Equation (1) to the temperature dependence data of gas permeance in Figure 14 for TiO_2_-ZrO_2_-MG and TiO_2_-ZrO_2_-EF membranes, are plotted as a function of the molecular kinetic diameter in Figure 15.

Judging from the two *k*_0_-plot lines shown in Figure 15a for the TiO_2_-ZrO_2_-MG membrane, the membrane had mainly two types of effective pores for permeation. The size of the first type was approximately 0.39 nm and that of the other seemed greater than 0.6 nm. The smaller molecules He, CO_2_ and N_2_ dominantly permeated the smaller 0.39 nm pores at high temperatures, while, at lower temperatures, permeance through the larger pores more significantly contributed to the overall permeance. The larger CF_4_ (0.47 nm) molecule permeated only the larger pores at all temperatures. The smaller pores originated from the organic materials coexistent with the TiO_2_-ZrO_2_ network pores and the larger pores seemed to be a type of grain boundary defect.

All the gas molecules passed through the small (0.46 nm) pores of the TiO_2_-ZrO_2_-EF membrane, as shown in Figure 15b. CO_2_ had an attractive interaction with the EF membrane, which seemed to be due to a combination of the intramolecular polarity of CO_2_ and the polar groups that originated from the aromatic organic chelating ligand. A mean pore diameter of 0.46 nm might also have contributed to this affinity during the permeation of CO_2_, which has a size of 0.33 nm. In addition, EF is known to form a dimer [46,47], which can fill the grain boundary pores for the formation of microporous membranes with fewer pinholes.

As a result, we successfully prepared two different types of gas permselective membranes by using aOCLs, as summarized in Table 4.

## 4. Conclusions

In this study, we prepared TiO_2_-ZrO_2_-aOCL composite membranes using methyl gallate (MG) and ethyl ferulate (EF), which are aromatic organic chelating ligands, and we investigated the gas permeation characteristics. In order to evaluate the materials used for the separation layer, dry gel and powder samples of the membrane material were prepared and TG and FT-IR measurements were performed. All organic substances were decomposed and removed by firing at 500 °C under air, but carbon compounds derived from the organic chelating ligands remained on the TiO_2_-ZrO_2_ composites when fired at 300 °C under a nitrogen atmosphere.

Powder samples were prepared under conditions where the organic chelating ligand remained and where the organic chelating ligand was decomposed and removed, and XRD and nitrogen adsorption measurements were performed. All the TiO_2_-ZrO_2_ composite materials could form an amorphous structure. From the nitrogen adsorption/desorption measurements, the powder sample with the aOCL had a larger amount of nitrogen adsorption and a larger BET-specific surface area than the powder sample prepared without the aOCL. On the other hand, when fired at 300 °C under a nitrogen atmosphere, the amount of nitrogen adsorbed by the powder sample with an aOCL was small due to the reduced pore volume by the residue of the thermally decomposed aOCL.

The TiO_2_-ZrO_2_ composite membrane prepared without the aOCL exhibited only Knudsen permeation selectivity, but the TiO_2_-ZrO_2_-MG and TiO_2_-ZrO_2_-EF membranes fired under nitrogen at 300 °C showed higher levels of molecular sieving permeation. The TiO_2_-ZrO_2_-MG membrane had relatively smaller pores with high permeability only for He, whereas the TiO_2_-ZrO_2_-EF composite membrane had a slightly larger mean pore size with fewer pinholes and exhibited molecular sieving permeation for molecules that ranged from 0.3–0.5 nm. It was suggested that gas separation membranes with different pore structures could be prepared depending on the organic chelating ligand used. J. Sunarso et al. reported that He permeance of about 10^−6^ mol/(m^2^ s Pa) is required for a He recovery process with a ceramic membrane [48]. The performance of the membranes developed in this study almost satisfies this requirement and is considered to be highly practical. The TiO_2_-ZrO_2_-MG membrane is expected to be applied to He/CH_4_ separation, and the TiO_2_-ZrO_2_-EF membrane is, by taking advantage of its high permeability, expected to be applied to the separation of He or H_2_ from larger molecules such as organic hydride. It is desired to develop a TiO_2_-ZrO_2_-aOCL membrane having a controlled pore size according to the separation target by selecting an appropriate organic chelate ligand and membrane preparation conditions. Although the performance of TiO_2_-ZrO_2_-MG and TiO_2_-ZrO_2_-EF membranes are inferior to silica-based membranes and MOF membranes, when compared to previously reported organic polymer-based membranes, it is close to the upper limit of the trade-off relationship between permeability and selectivity. Specifically, the He permeability is as high as about 10^−6^ mol/(m^2^ s Pa); therefore, it is expected to be practically used as a ceramic-based stable He recovery membrane by establishing mass production technology and suppressing the manufacturing cost.

We previously reported that TiO_2_-ZrO_2_-isoeugenol (ISOH) composite membranes show good gas permselectivity for CO_2_ and CH_4_ [33]. All the OCLs of ISOH, MG, and EF have an aromatic ring in the chemical structure and the benzene ring could effectively remain in the TiO_2_-ZrO_2_ amorphous structure after firing under nonoxidation conditions to form an inorganic/organic nano-hybrid porous composite material. These aromatic organic chelating ligands would be useful in preparing TiO_2_-ZrO_2_-based microporous gas separation membranes.

## Figures and Tables

**Table 1:** Compositions of chemical reagents and solvents for preparation of TiO_2_-ZrO_2_(-aOCL) sols.

wt%	Sol Type	Chelating Ligand	1-PrOH (g)	ZrTB (g)	TiTP (g)	H_2_O (g)	HCl (g)	Chelating Ligand (g)
1.0	Polymer	*Non*	58.70	0.27	0.33	0.10	0.60	0
1.0	Polymer	MG	58.42	0.27	0.33	0.10	0.60	0.28
1.0	Polymer	EF	58.47	0.27	0.33	0.10	0.60	0.23

**Table 2:** Adsorbed amounts and BET surface areas measured by N_2_ adsorption/desorption.

Sample	Adsorbed Amount (cm^3^/g)	BET Surface Area (m^2^/g)
TiO_2_-ZrO_2_	27.6	34.9
TiO_2_-ZrO_2_-MG	39.9	72.2
TiO_2_-ZrO_2_-EF	50.0	104

**Table 3:** Characteristics of the intermediate layer membrane.

Intermediate Layer *for*	Mean Pore Size (nm)	Leakage Ratio (Kelvin Diameter)	Dry N_2_ Permeance (10^−6^ mol/(m^2^ s Pa))
TiO_2_-ZrO_2_-MG	0.5–1	0.41% (9.64 nm)	4.91
TiO_2_-ZrO_2_-EF	0.5–1	0.47% (9.64 nm)	4.97

**Table 4:** Gas permeance ratio at 200 °C.

Membrane	Permeance Ratio [–]			
	He/CF_4_	CO_2_/N_2_	He/N_2_	He/CH_4_
TiO_2_-ZrO_2_	11.7	1.26	4.73	3.88
TiO_2_-ZrO_2_-MG	209	1.66	61.7	111
TiO_2_-ZrO_2_-EF	163	2.47	10.6	13.6

**Table 5:** Apparent activation energy for gas permeation of TiO_2_-ZrO_2_-MG and TiO_2_-ZrO_2_-EF membranes.

Membrane	*E*_P_ in Equation (1) [kJ/mol]				
	He	CO_2_	N_2_	CH_4_	CF_4_
TiO_2_-ZrO_2_-MG	9.9	6.7	7.1	−1.0	−4.5
TiO_2_-ZrO_2_-EF	3.9	−8.5	−3.5	−2.5	−9.0

## Supplementary Materials

The Supporting Information are available online at https://www.mdpi.com/2077-0375/10/12/388/s1.
