# Supplementary material for: Gas Permeation Characteristics of TiO2-ZrO2-Aromatic Organic Chelating Ligand (aOCL) Composite Membranes
Source: Membranes (Basel). 2020 Dec 1;10(12):388. doi: 10.3390/membranes10120388 (PMC7760951; doi:10.3390/membranes10120388)
Supplement: Supplementary file 1 [file membranes-10-00388-s001.pdf]

membranes

Supporting Information

Gas Permeation Characteristics of TiO_2-ZrO_2-Aromatic Organic Chelating Ligand (aOCL) Composite Membranes

Takashi Tachibana [1], Tomohisa Yoshioka [1,*], Keizo Nakagawa [1], Takuji Shintani [1], Eiji Kamio [2] and Hideto Matsuyama [2]

[1] Research Center for Membrane and Film Technology, Graduate School of Science, Technology, and Innovation, Kobe University, 1-1 Rokkodai, Nada, Kobe 657-8501, Japan; 187p106p@stu.kobe-u.ac.jp (T.T.); k.nakagawa@port.kobe-u.ac.jp (K.N.); shintani@port.kobe-u.ac.jp (T.S.)
[2] Research Center for Membrane and Film Technology, Department of Chemical Science and Engineering, Kobe University, 1-1 Rokkodai, Nada, Kobe 657-8501, Japan; e-kamio@people.kobe-u.ac.jp (E.K.); matuyama@kobe-u.ac.jp (H.M.)
* Correspondence: tom@opal.kobe-u.ac.jp; Tel.: +81-78-803-6299

1. Aromatic Organic Chelating Ligand (aOCL) Used.

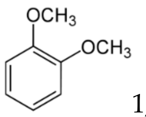

1,2-Dimethoxybenzene (DMB)

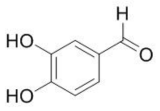

3,4-Dihydroxybenzaldehyde (DHB)

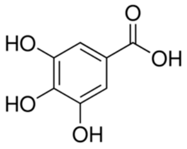

Gallic Acid Monohydrate (GAH)

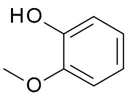

Guaiacol (GAC)

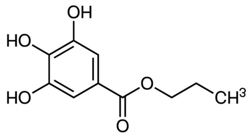

Propyl Gallate (PG)

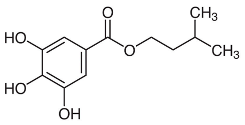

Isoamyl Gallate (IG)

Previous Work:

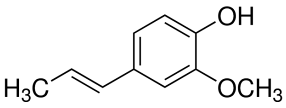

Isoeugenol (ISOH)

Fukumoto, T.; Yoshioka, T. *et al.* *J. Membr. Sci.* **2014**, *461*, 96–105. doi:10.1016/j.memsci.2014.02.031.

This Work:

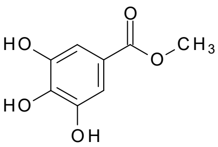

Methyl gallate (MG)

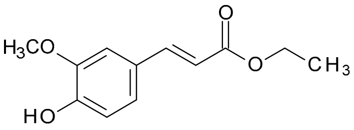

Ethyl ferulate (EF)

1.1. 1,2-Dimethoxybenzene (DMB)

Figure S1 shows the change in the solution appearance during the TiO_2-ZrO_2 sol preparation procedure illustrated in Figure 2 for the case of DMB instead of MG or EF.

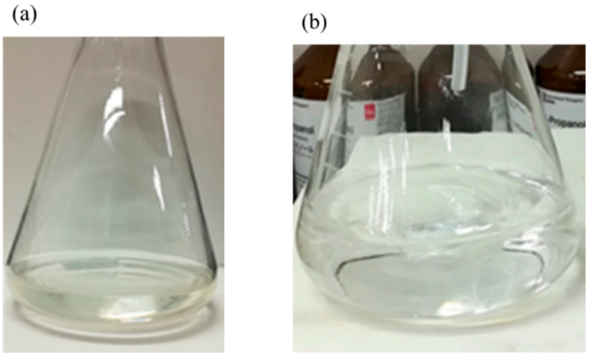

Figure S1. TiO_2-ZrO_2-DMB; (**a**) Before addition of DMB, (**b**) After addition of DMB.

We could see no change in color of the solution which indicated no coordination of aOCL to metal elemnt occurred. The charge of O in –OCH_3 group was lower than that in –OH group and it might make difficult to coordinate to the metal alkoxide.

1.2. 3,4-Dihydroxybenzaldehyde (DHB)

Figure S2 shows the change in the solution appearance during the TiO_2-ZrO_2 sol preparation procedure illustrated in Figure 2 for the case of DHB.

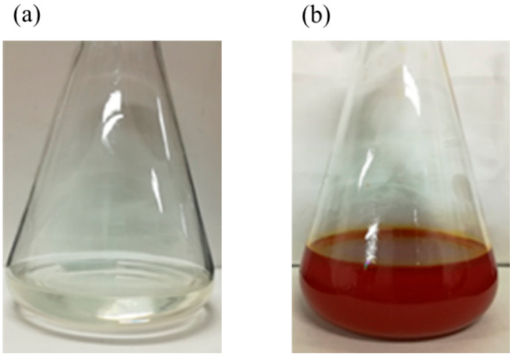

Figure S2. TiO_2-ZrO_2-DHB; (**a**) Before addition of DHB, (**b**) After addition of DHB.

We could see change in color of the solution to red, however, we also found some precipitate. Therefore, we have judged it could not be suitable for coating.

1.3. Gallic Acid Monohydrate (GAH)

Figure S3 shows the change in the solution appearance during the TiO_2-ZrO_2 sol preparation procedure illustrated in Figure 2 for the case of GAH.

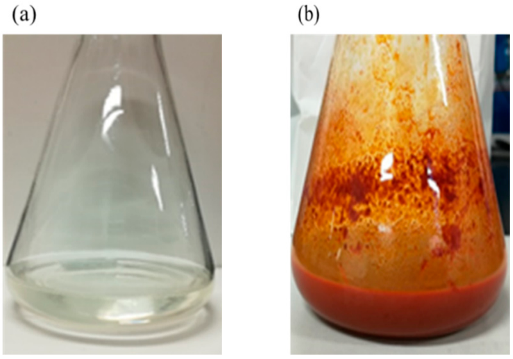

Figure S3. TiO_2-ZrO_2-GAH; (**a**) Before addition of GAH, (**b**) After addition of GAH.

We could found some precipitate as well as the case of DHB. Therefore, we have judged it could not be suitable for coating. Judging from the fact that addition of DHB also caused precipitate, an aOCL having an aldehyde or carboxyl group in the side chain with hydroxyl group might cause polymerization among organic chelating ligand as shown in Figure S4.

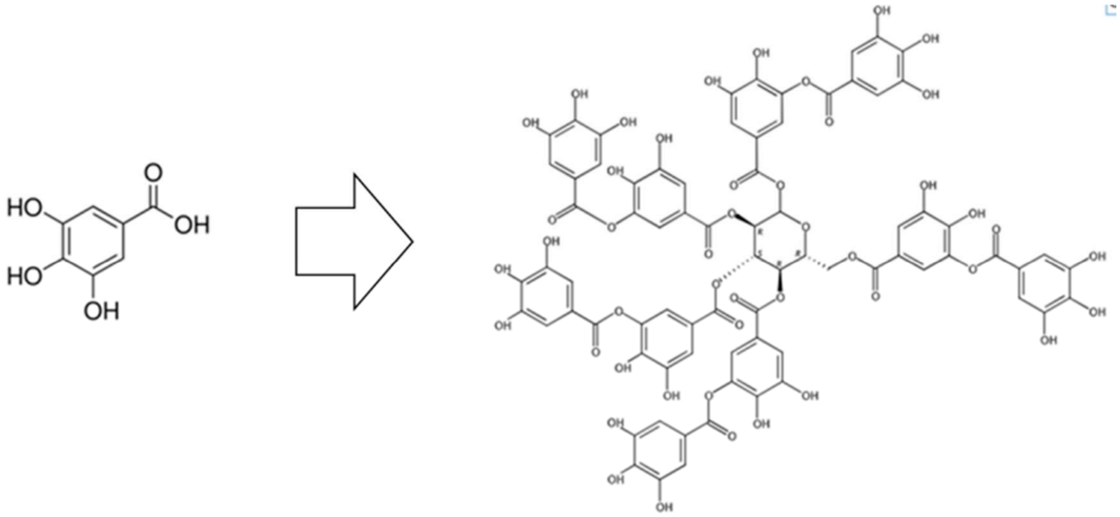

Figure S4. Polymerization of Gallic Acid Monohydrate.

1.4. Guaiacol (GAC)

Figure S5 shows the change in the solution appearance during the TiO_2-ZrO_2 sol preparation procedure illustrated in Figure 2 for the case of GAC. We could see change in color of the solution to yellow, and a clear solution was successfully obtained.

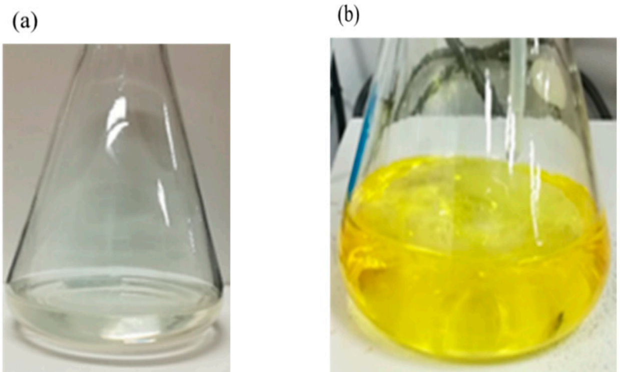

Figure S5. TiO_2-ZrO_2-GAC; (**a**) Before addition of GAC, (**b**) After addition of GAC.

Figure S6 shows the FT-IR spectrum of TiO_2-ZrO_2-GAC gels calcined at different temperatures under N_2. The peak around 1600 cm^{-1} observed for non-calcined dried gel corresponded to the peak of C - O stretching oscillations derived from aromatic rings. The peak disappeared after calcination at temperatures above 300 °C under nitrogen atmosphere, suggesting that no guaiacol remained on the material after firing at these temperatures.

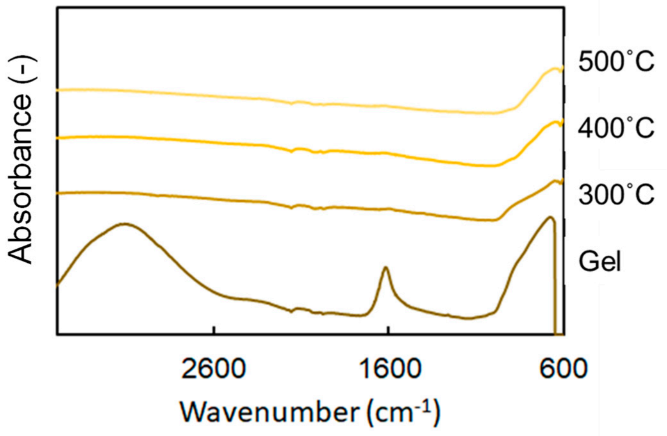

Figure S6. FT-IR spectrum of TiO_2-ZrO_2-GAC gels calcined under N_2.

Figure S7. shows the Kinetic diameter dependency of gas permeance at 200 °C of TiO_2-ZrO_2-GAC membrane. The permeance ratio of each gas molecule of the TiO_2-ZrO_2-GAC composite membrane was nearly similar with that of its intermediate layer. As predicted from the FT-IR results, the thermal stability of GAC was low and could be completely decomposed during the fireing for membrane preparation, resulting in too large pore size to show molecular sieving performance.

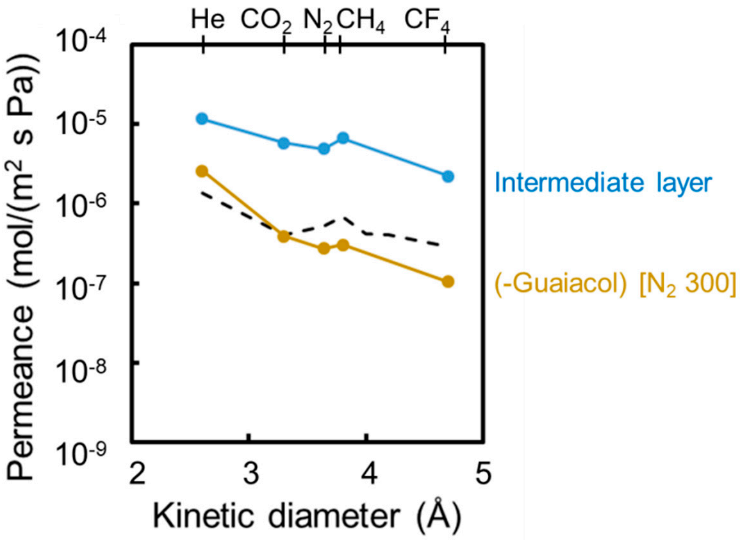

Figure S7. Kinetic diameter dependency of gas permeance at 200°C of TiO_2-ZrO_2-GAC membrane.

1.5. Propyl Gallate (PG)

Figure S8 shows the change in the solution appearance during the TiO_2-ZrO_2 sol preparation procedure illustrated in Figure 2 for the case of PG. We could see change in color of the solution to dark red as the case of ISOH [S2], and a clear solution was successfully obtained.

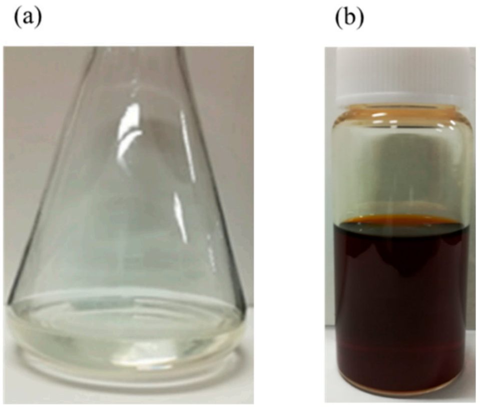

Figure S8. TiO_2-ZrO_2- PG; (**a**) Before addition of PG, (**b**) After addition of PG.

Figure S9 shows the FT-IR spectrum of TiO_2-ZrO_2-PG gels calcined at different temperatures under N_2. Some peaks corresponding to C=O, C–O, and C=C stretching oscillations could be observed for the gel fired at 300 °C under nitrogen atmosphere, suggesting that organic matters originated PG might be remained on the material after firing at this temperature.

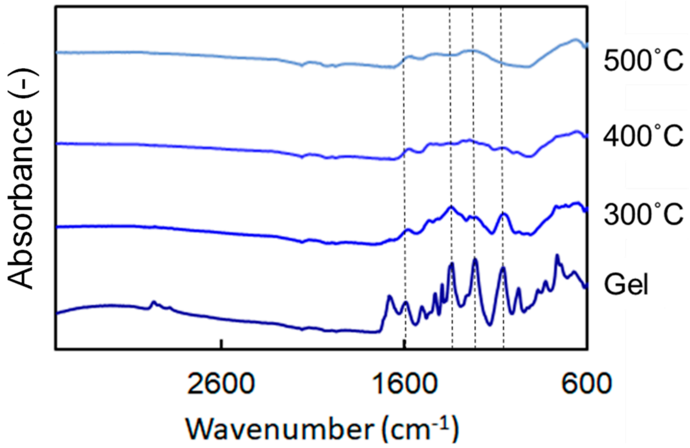

Figure S9. FT-IR spectrum of TiO_2-ZrO_2-PG gels calcined under N_2.

Figure S10. shows the Kinetic diameter dependency of gas permeance at 200 °C of TiO2-ZrO2-PG membrane. The permeance ratio of each gas molecule of the TiO2-ZrO2-PG composite membrane was nearly similar with that of its intermediate layer as well as TiO2-ZrO2-GAC membrane. Since –C_3H_7 group was easily decomposed at temperatures above 200 °C, it might form larger pores as shown schematically in Figure S11, resulting in reduced molecular sieving effect.

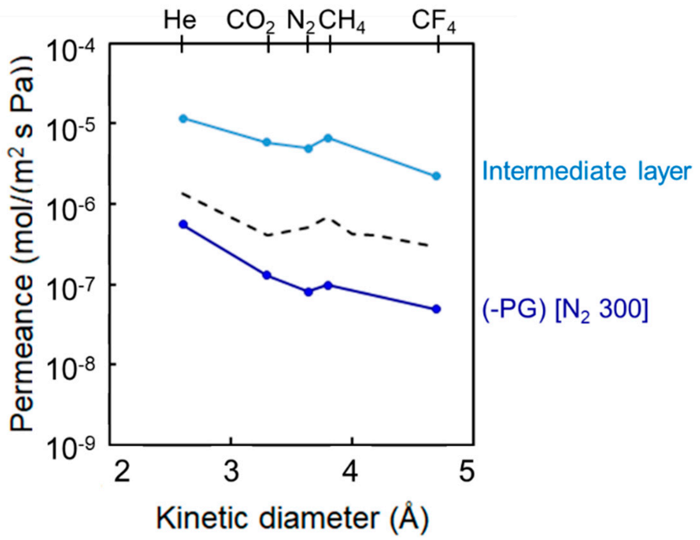

Figure S10. Kinetic diameter dependency of gas permeance at 200°C of TiO_2-ZrO_2-PG membrane.

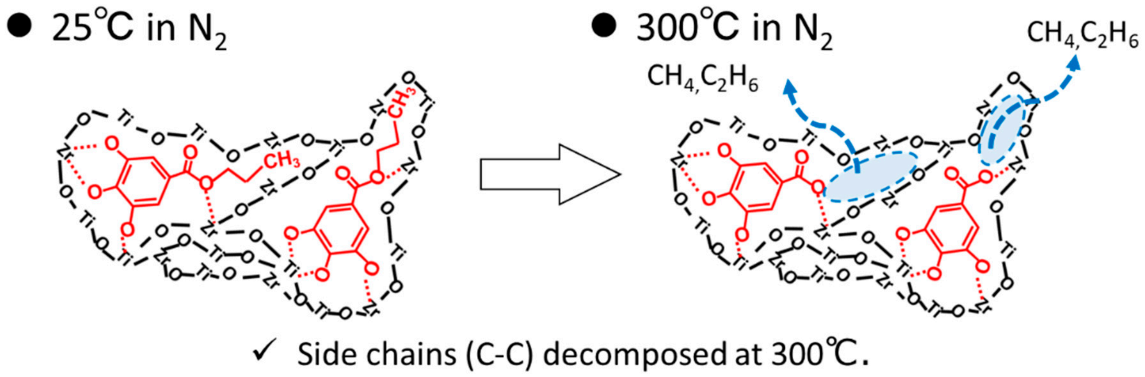

Figure S11. Schematic image of possible thermal decomposition of PG.

1.6. Isoamyl Gallate (IG)

Figure S12 shows the change in the solution appearance during the TiO_2-ZrO_2 sol preparation procedure illustrated in Figure 2 for the case of IG. We could see change in color of the solution to red, and a clear solution was successfully obtained.

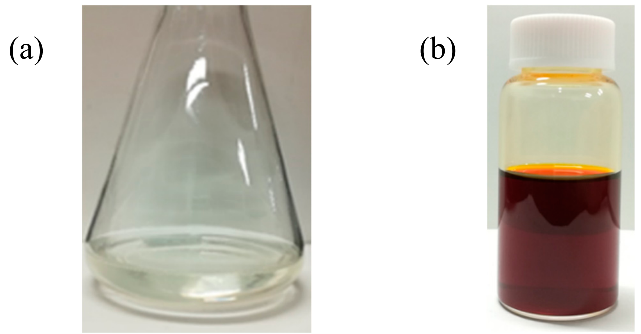

Figure S12. TiO_2-ZrO_2-IG; (**a**) Before addition of IG, (**b**) After addition of IG.

Figure S13 shows the FT-IR spectrum of TiO_2-ZrO_2-IG gels calcined at different temperatures under N_2. Some peaks corresponding to C=O, C-O, and C=C stretching oscillations could be observed for each gel fired at different temperatures, but were reduced with elevating the firing temperature.

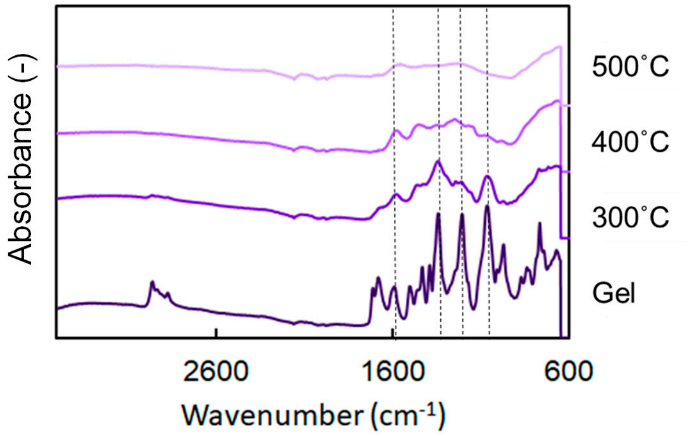

Figure S13. FT-IR spectrum of TiO_2-ZrO_2-IG gels calcined under N_2.

Figure S14 shows the Kinetic diameter dependency of gas permeance at 200 °C of TiO_2-ZrO_2-IG membrane. The permeance ratio of CO_2/N_2 seemed to be obviously improved in comparison with that of the intermediate layer and was greater than that of –GAC and –PG membranes. However, decomposition of side chain such as isoamyl group of IG could possibly form too large voids for effective gas separation. As the result, the gas permeance ratios might still be poor.

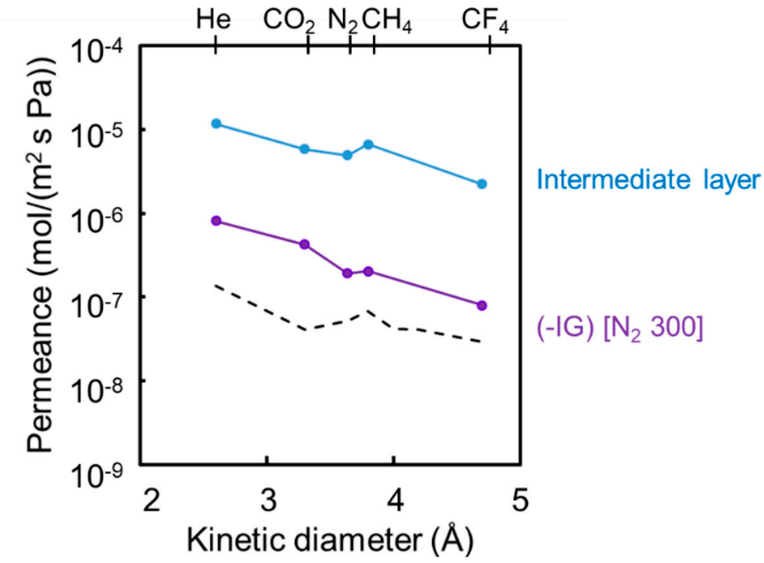

Figure S14. Kinetic diameter dependency of gas permeance at 200°C of TiO_2-ZrO_2-PG membrane.

1.7. Summary of TiO_2-ZrO_2-aOCL Composite Membranes

Helium gas permeance and permeance selectivity (He/CF_4) observed for several TiO_2-ZrO_2-aOCL composite membranes were summarized in Figure S15. In the main text, we have discussed the membrane performance and membrane porous structures of TiO_2-ZrO_2-MG and TiO_2-ZrO_2-EF membranes.

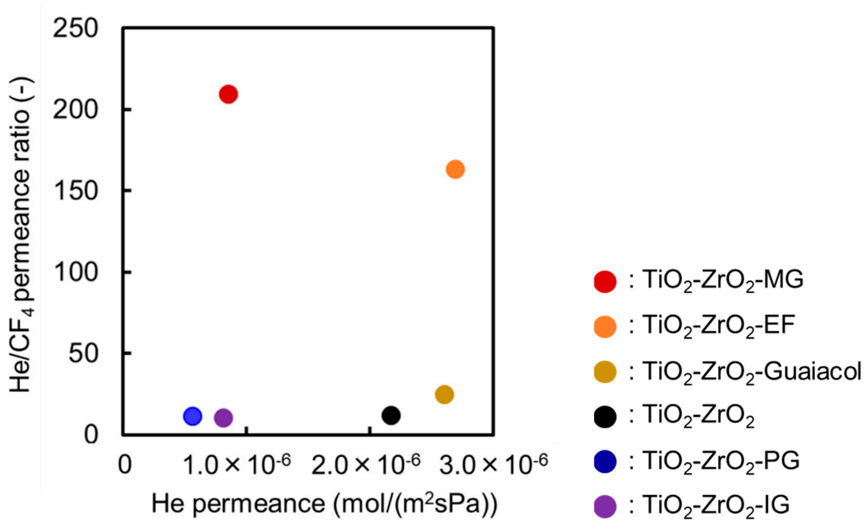

Figure S15. Performance of various TiO_2-ZrO_2-aOCL composite membranes.
